# Impact of neoadjuvant relugolix on patient-reported sexual function and bother

**DOI:** 10.3389/fonc.2024.1377103

**Published:** 2024-04-11

**Authors:** Jessica Y. Hsueh, Lindsey Gallagher, Min Ji Koh, Shaine Eden, Sarthak Shah, Markus Wells, Malika Danner, Alan Zwart, Marilyn Ayoob, Deepak Kumar, Paul Leger, Nancy A. Dawson, Simeng Suy, Rachel Rubin, Sean P. Collins

**Affiliations:** ^1^ Department of Radiation Medicine, MedStar Georgetown University Hospital, Washington, DC, United States; ^2^ Systems Medicine Program, Department of Biochemistry and Molecular & Cellular Biology, Georgetown University Medical Center, Washington, DC, United States; ^3^ Biotechnology Research Institute, North Carolina Central University, Durham, NC, United States; ^4^ Department of Oncology, Lombardi Comprehensive Cancer Center, MedStar Georgetown University Hospital, Washington, DC, United States; ^5^ Department of Urology, MedStar Georgetown University Hospital, Washington, DC, United States

**Keywords:** prostate cancer, stereotactic body radiation therapy (SBRT), relugolix, sexual function, bother, androgen deprivation therapy (ADT)

## Abstract

**Introduction:**

Sexual function following local treatment for prostate cancer is an important quality of life concern. Relugolix is a novel oral GnRH receptor antagonist used in combination with radiation therapy in the treatment of unfavorable prostate cancer. It has been shown to achieve rapid and profound testosterone suppression. As a result, these very low testosterone levels may impact both sexual functioning and perceptions. This prospective study sought to assess neoadjuvant relugolix-induced sexual dysfunction prior to stereotactic body radiation therapy (SBRT).

**Methods:**

Between March 2021 and September 2023, 87 patients with localized prostate cancer were treated with neoadjuvant relugolix followed by SBRT per an institutional protocol. Sexual function and bother were assessed via the sexual domain of the validated Expanded Prostate Index Composite (EPIC-26) survey. Responses were collected for each patient at pre-treatment baseline and after several months of relugolix. A Utilization of Sexual Medications/Devices questionnaire was administered at the same time points to assess erectile aid usage.

**Results:**

The median age was 72 years and 43% of patients were non-white. The median baseline Sexual Health Inventory for Men (SHIM) score was 13 and 41.7% of patients utilized sexual aids prior to relugolix. Patients initiated relugolix at a median of 4.5 months (2-14 months) prior to SBRT. 95% and 87% of patients achieved effective castration (≤ 50 ng/dL) and profound castration (< 20 ng/dl) at SBRT initiation, respectively. Ability to have an erection, ability to reach orgasm, quality of erections, frequency of erections, and overall sexual function significantly declined following relugolix. There was a non- significant increase in sexual bother.

**Discussion:**

In concordance with known side effects of androgen deprivation therapy (ADT), neoadjuvant relugolix was associated with a significant decline in self-reported sexual function. However, patients indicated only a minimal and non-significant increase in bother. Future investigations should compare outcomes while on relugolix directly to GnRH agonist-induced sexual dysfunction.

## 1 Introduction

National guidelines currently recommend radiation therapy (RT) and androgen deprivation therapy (ADT) as the standard of care for unfavorable intermediate and high risk prostate cancer (1). Multiple trials have demonstrated improved prostate-cancer specific mortality with the addition of ADT to external beam radiation therapy (EBRT) (2, 3). As with EBRT, early data suggests that the addition of ADT to stereotactic body radiation therapy (SBRT) for unfavorable prostate cancer may also reduce local cancer persistence and biochemical recurrence (4, 5). Unfortunately, ADT combined with RT remains underutilized as a treatment modality, possibly due to bothersome side effects such as sexual dysfunction (6).

Sexual function declines rapidly following ADT but generally returns following testosterone recovery (7). The etiology of ADT-induced sexual dysfunction involves decreased libido and penile contractility impairment (8). Patients with ADT-induced sexual dysfunction report a decrease in the reliability, quality and frequency of erections, ability to reach orgasm and overall ability to function sexually (9). More extended durations of ADT can lead to increasingly severe and persistent side effects (10). Patient characteristics such as advanced age, obesity, partner status, baseline erectile dysfunction (ED), and pretreatment sexual aid usage may increase the risk of sexual impairment (10). Treatment-related factors, including castration level, may also contribute to the incidence and severity of sexual dysfunction.

Injectable GnRH receptor agonists like leuprolide are commonly utilized for testosterone suppression in prostate cancer patients. Following initial administration, testosterone levels and sexual function undergo a slow decline. Eventual testosterone recovery and return of sexual function has been established as unpredictable. The median testosterone recovery time after discontinuing ADT is often prolonged, and a considerable proportion of men may never achieve normal testosterone levels (10–12). Several factors, such as older age, lower baseline testosterone levels, and longer duration of ADT, have been associated with slower testosterone recovery (11–13).

Relugolix is a new oral GnRH receptor antagonist that has been shown to achieve rapid and profound testosterone suppression (<20 ng/dL; 0.7 nmol/L) with quicker testosterone recovery following discontinuation (14). The randomized phase 3 HERO trial, which compared leuprolide to relugolix, found that relugolix was superior in achieving sustained castration (15). Profound castration may adversely alter sexual functioning and perceptions. It is largely unknown whether prostate cancer patients receiving RT are significantly troubled by short-term relugolix-induced sexual dysfunction. Few studies have elucidated the impact of relugolix on sexuality. While analysis from the HERO trial showed that patients on relugolix experienced decline in sexual function and activity, the questionnaire utilized was limited in scope (16). We sought to characterize the degree to which neoadjuvant relugolix affects sexual function and bother prior to SBRT.

## 2 Materials and methods

We conducted a prospective investigation of patients diagnosed with intermediate to high-risk prostate cancer at MedStar Georgetown University Hospital (IRB 12-1775). Data was extracted from medical records to obtain details on age, race, partner status, body mass index (BMI), Gleason score, and pretreatment Sexual Health Inventory for Men (SHIM) score. Risk groups were established using the D’Amico classification.

### 2.1 Drug treatment

Neoadjuvant relugolix was initiated at least two months prior to SBRT. A loading dose of 360 mg was given on the first day, with a 120 mg oral dose taken daily at approximately the same time each day. Patients were educated prior to treatment that sexual dysfunction is a known side effect of relugolix but should resolve after discontinuation in conjunction with testosterone recovery.

### 2.2 Sexual function and bother follow-up and assessment

Potency was defined as firm enough for intercourse with or without sexual aids, while sexual activity was defined as the ability to have an erection firm enough for masturbation and foreplay. Sexual function and bother were assessed for the prior month via the sexual domain of the validated Expanded Prostate Index Composite (EPIC-26) survey, which was collected for each patient at pre-treatment baseline and one hour prior to SBRT initiation. The EPIC-26 sexual domain includes five questions related to function (ability to have an erection, ability to have an orgasm, erection quality, erection frequency and overall sexual function) and one overall bother question. A Utilization of Sexual Medications/Devices questionnaire was also administered at the same time points to assess for the use of erectile aids, including phosphodiesterase-5 (PDE-5) inhibitors, suppositories, vacuum erection devices, penile injection therapy, and penile prostheses. Serum total testosterone levels were obtained concurrently with the administration of both EPIC-26 and Utilization of Sexual Medications/Devices questionnaires.

Individual EPIC-26 responses were scored from 0 to 100, with higher scores reflecting improved function/less bother. Responses to individual questions were grouped into clinically relevant categories. Overall sexual bother scores were organized into different categories, ranging from no problem to big problem. Wilcoxon rank sum test was used to examine changes before and after relugolix treatment. Statistical significance was determined with the McNemar test (p < 0.05). Clinical significance was assessed via minimally important difference (MID), calculated by 0.5 of the standard deviation at baseline.

## 3 Results

### 3.1 Patient demographics and characteristics

Patient characteristics are outlined in **Table 1**. Between March 2021 and September 2023, 87 patients with localized prostate cancer were treated with neoadjuvant relugolix followed by SBRT. The median age of our cohort was 72 years (range: 49-87). 56% patients were Caucasian, 32% patients were African American, and 11% patients identified as another race. Most were married/partnered (75%). 24% were obese with a BMI ≥30 kg/m (2). 80% of patients had intermediate risk disease per D’Amico classification. 78% had ED prior to treatment (baseline SHIM ≤ 21) with a median baseline SHIM of 13 (range: 1-25). Patients initiated relugolix at a median of 4.4 months prior to SBRT (range: 2-14.4). 95% and 87% of patients achieved effective castration (≤ 50 ng/dL) and profound castration (≤ 20 ng/dL) at SBRT initiation, respectively (**Table 2**).

**Table 1:** Demographic and clinical characteristics of our patient cohort.

Characteristics	No (%) (n = 87)
Age (y), Median (IQR) <60 60-69 70-79 >80	72 (66-76) 6 (7) 34 (39) 37 (43) 10 (11)
Race White Black Other	49 (56) 28 (32) 10 (11)
Partner Status Partnered Non-Partnered	65 (75) 22 (25)
Gleason Score 3 + 3 = 6 3 + 4 = 7 4 + 3 = 7 4 + 4 = 8 4 + 5 = 9	6 (7) 20 (23) 46 (53) 14 (16) 1 (1)
Risk Group Intermediate High	69 (80) 17 (20) *(n = 86)*
BMI (kg/m2), Median (IQR) <18.5 18.5-24.9 25-29.9 30-34.9 35-39.9 40-44.9	27 (25-30) 0 23 (28) 39 (48) 15 (18) 4 (5) 1 (1) *(n = 82)*
Prostate Volume (cc), Median (IQR)	37 (28-50) *(n = 86)*
Pre-treatment SHIM, Median (IQR) 1-7 Severe ED 8-11 Moderate ED 12-16 Mild-to-moderate ED 17-21 Mild ED 22-25 No ED	13 (6-20) 26 (31) 12 (14) 11 (13) 17 (20) 18 (21) *(n = 84)*

**Table 2:** Testosterone levels at SBRT initiation.

Characteristics	No (%) n = 87
Effective Castration (Testosterone ≤50ng/dL) Yes No Profound Castration (Testosterone ≤20ng/dL) Yes No	83 (95) 4 (5) 76 (87) 11 (13)

Effective castration was defined as testosterone levels < 50 ng/dL, while profound castration was defined as testosterone levels < 20 ng/dL.

### 3.2 Sexual function changes

**Figure 1:**
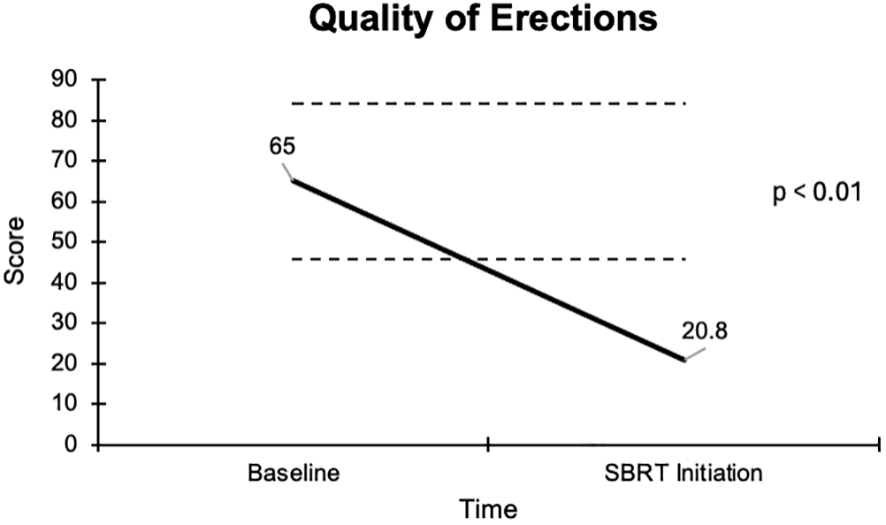
Graph of average baseline and SBRT initiation scores for erection quality.

The EPIC-26 sexual summary domain demonstrates a comprehensive and reliable assessment of a patient’s overall sexual function. Patient responses to the EPIC-26 survey are summarized in **Table 3**. After several months of relugolix, we found that patients experienced a decrease in sexual function in all EPIC-26 domains. Erection quality, an important element of sexual function, was assessed in question 9 of the EPIC-26 survey. Following neoadjuvant relugolix, potency declined from 46% to 10%, while sexual activity declined from 22% to 8%. There was a noticeable increase in patients who could not have any erections from 18% at baseline to 65% at SBRT initiation. The average score indicating quality of erections decreased from 65 to 20.8 for a -44.2 change, which was deemed statistically and clinically significant (**Figure 1**).

**Table 3:** Sexual functions and bother following neoadjuvant relugolix.

Characteristics	No. (%)		Change	Statistical Significance *(P* value^a^)	Clinical Significance (MID)
	Baseline	SBRT Initiation			
**Erection quality** Mean Score Firm enough for intercourse Masturbation and foreplay only Not firm for any sexual activity None at all	65 ± 8.5 36 (46) 17 (22) 12 (15) 14 (18)	20.8 ± 3.3 8 (10) 6 (8) 14 (18) 51 (65)	44.2	<0.01	+
**Ability to have an erection** Mean Score Very good-good Fair Poor, very poor to none	(n = 82) 46.6 ± 3.5 27 (33) 25 (30) 30 (37)	(n = 82) 17.4 ± 6.6 9 (11) 7 (9) 66 (80)	29.2	<0.01	+
**Ability to reach orgasm** Mean Score Very good-good Fair Poor, very poor to none	(n = 82) 52.7.± 34 36 (44) 21 (26) 25 (30)	(n = 82) 15.5 ± 7.4 8 (10) 6 (7) 66 (83)	37.2	<0.01	+
**Frequency of erections** Mean Score More than half-half the time Less than half the time	(n = 77) 55.1 ± 7.7 50 (65) 27 (35)	(n = 77) 19.6 ± 3.7 15 (19) 62 (81)	35.5	<0.01	+
**Overall sexual function** Mean Score Very good-good Fair Poor, very poor	(n = 77) 48.1.± 35.3 32 (42) 15 (19) 30 (39)	(n = 77) 17.6 ± 8.1 9 (12) 5 (6) 63 (82)	30.5	<0.01	+
**Overall sexual bother** Mean Score Big problem Moderate problem Small problem Very small problem No problem	(n = 78) 64.7± 36.1 31 (40) 15 (19) 10 (13) 13 (17) 9 (12)	(n = 78) 56.7 ± 41.4 28 (36) 11 (14) 10 (13) 9 (12) 20 (26)	8.0	0.06	−

^a^P values for each category were calculated using the McNemar test.

Each item represents a question from the EPIC-26 survey. Clinical significance is denoted by + (clinical significance present) or – (clinical significance absent).

**Figure 2:**
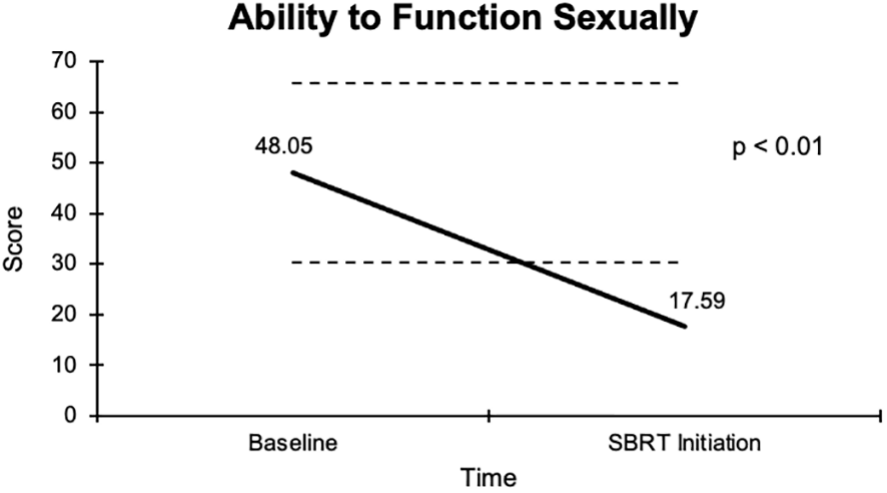
Graph of average baseline and SBRT initiation scores for the ability to function sexually.

**Figure 3:**
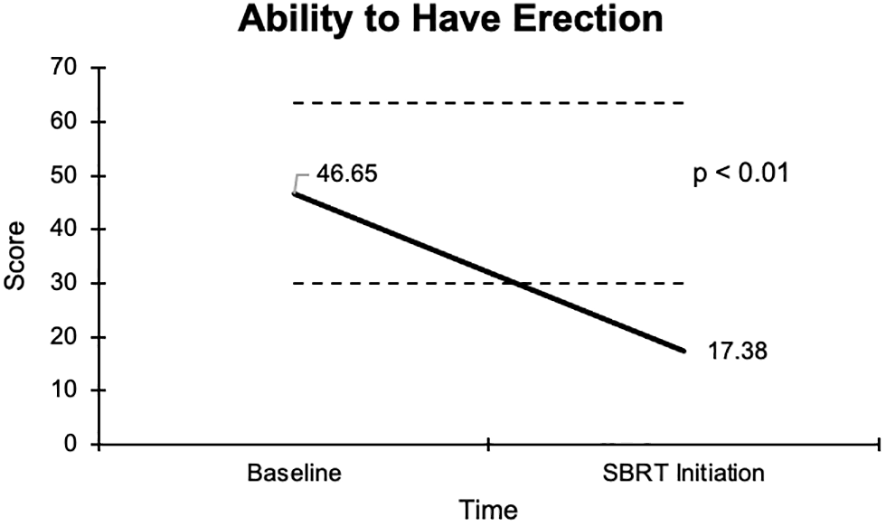
Graph of average baseline and SBRT initiation scores for the ability to have an erection.

**Figure 4:**
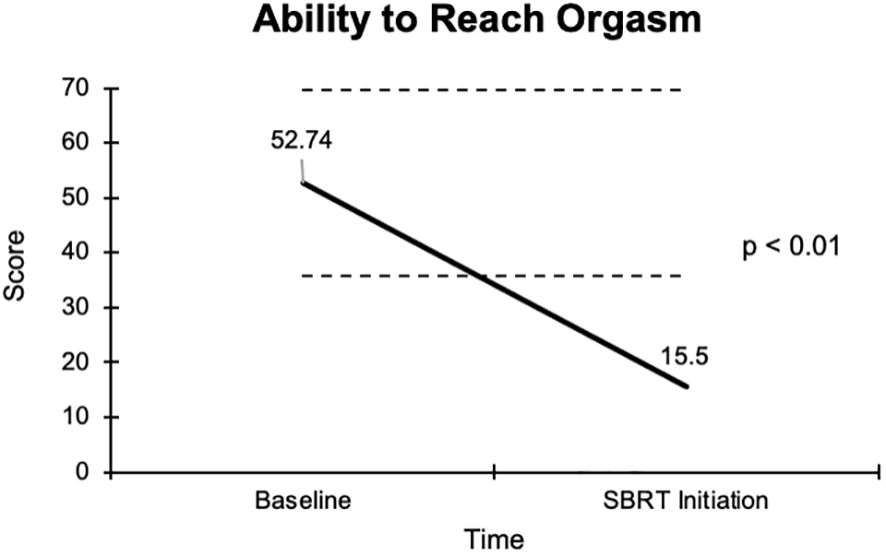
Graph of average baseline and SBRT initiation scores for the ability to reach orgasm.

**Figure 5:**
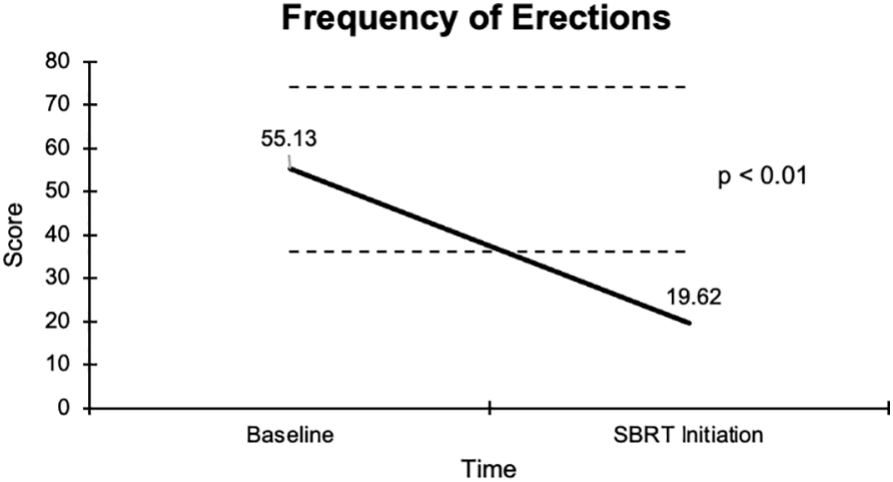
Graph of average baseline and SBRT initiation scores for erection frequency.

The overall sexual function score declined from 48.1 at baseline to 17.6 at the start of SBRT, with a -30.5 change from baseline (**Figure 2**). This decline was both statistically (p <0.01) and clinically significant (MID = 17.65). There was a -29.2 change for the ability to have an erection from a 46.6 baseline score to 17.4 (**Figure 3**), -37.2 change for ability to reach orgasm from a baseline score of 52.7 to 15.5 (**Figure 4**), and a -35.5 change for erection frequency from a 55.1 baseline score to 19.6 (**Figure 5**). Similarly, these findings were all statistically and clinically significant.

**Figure 6:**
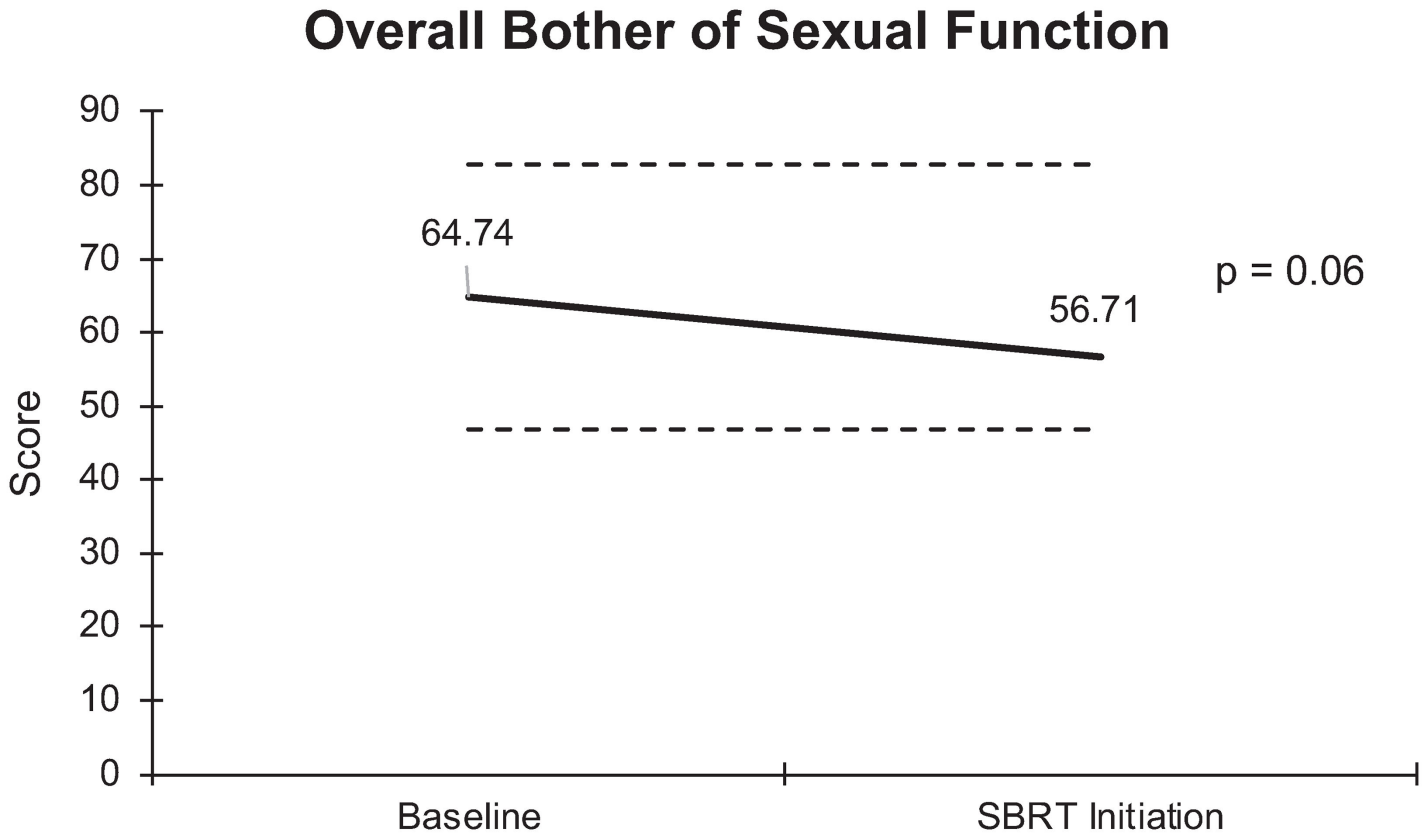
Graph of average baseline and SBRT initiation scores for overall bother of sexual function.

Unlike the significant changes observed in sexual functioning, there was no significant increase in sexual bother with relugolix. At baseline, 59% of the cohort reported feeling that their sexual dysfunction was a moderate to big problem. However, only 50% of patients felt similarly after relugolix. An average baseline EPIC-26 sexual bother score of 64.7 decreased to 56.7 after several months of relugolix (**Figure 6**). This reduction was neither statistically (p = 0.06) nor clinically significant (MID = 18.05).

### 3.3 Sexual aid use

**Table 4** summarizes sexual aid usage. Out of 84 patients surveyed, 30% utilized sexual aids prior to initiation of relugolix. The most commonly utilized treatment included PDE-5 inhibitors. Sexual aid usage declined to 13% while on relugolix. No patients utilized Muse, penile injections or vacuum devices at the time of SBRT initiation.

**Table 4:** Sexual aid utilization following neoadjuvant relugolix.

Characteristics	No. (%)		*P* value^a^
	Baseline (n = 84)	SBRT Initiation (n = 84)	
**Viagra** Have not tried Tried, but not helpful Helped, but not using now Helped, use sometimes Helped, use always	51 (61) 5 (6) 7 (8) 10 (12) 11 (13)	58 (69) 7 (8) 8 (10) 5 (6) 6 (7)	<0.01
**Muse suppository** Have not tried Tried, but not helpful Helped, but not using now Helped, use sometimes Helped, use always	84 (100) 0 0 0 0	84 (100) 0 0 0 0	–
**Penile injection therapy** Have not tried Tried, but not helpful Helped, but not using now Helped, use sometimes Helped, use always	83 (99) 0 0 1 (1) 0	83 (99) 1 (1) 0 0 0	>0.99
**Vacuum erection device** Have not tried Tried, but not helpful Helped, but not using now Helped, use sometimes Helped, use always	83 (99) 1 (1) 0 0 0	83 (99) 1 (1) 0 0 0	–

^a^ P values for each category were calculated using the McNemar test.

## 4 Discussion

It is well-established that ADT decreases libido and induces sexual dysfunction secondary to testosterone suppression. In a 2021 meta-analysis of nine studies, Corona et al. found that ADT resulted in an almost six-fold increased risk of reduced libido and three-fold increased risk of ED in prostate cancer patients (17). Similarly, an investigation within the Prostate Cancer Outcomes Study found that over two-thirds of ADT patients experienced loss of potency after treatment initiation (18). The same group demonstrated that 85.8% prostate cancer patients on ADT faced complete loss of erectile function, which was significantly higher than patients who received other treatments, such as prostatectomy and radiation therapy (19).

ADT-induced sexual dysfunction remains a persistent obstacle in treating patients with unfavorable prostate cancer. In this study, we employed the EPIC-26 questionnaire’s sexual function domain to assess changes in ED severity, orgasm quality, and subjective sexual bother. Our study supports previously validated findings and demonstrates a significant decline in all sexual function domains with relugolix, adding to the current literature on the toxicity of relugolix (16). This investigation is unique in the fact that all patients were exclusively treated with relugolix, in contrast to other studies that predominantly focus on GnRH agonists for ADT.

Sexual bother may be more imperative than sexual function in assessment of quality of life. While the majority of men receiving ADT will experience sexual impairment to some extent, there is wide variance in degree of bother (20). Our results provide insight into patient bother and interestingly show a non-significant increase following several months of relugolix. Overall, individuals did not express substantial distress about their sexual dysfunction. There are various reasons why patients may experience minimal bother, with previous studies revealing a weak correlation between sexual function and bother (20). Bother has been found to be more pronounced in cases of short-term ADT, particularly among younger patients and individuals with higher baseline sexual function (20, 9. 22). Given that older patients are more likely to exhibit diminished sexual function at baseline, the effects of ADT on intimacy may be less impactful, resulting in decreased bother. It is pertinent to emphasize that the median age of our patient cohort was 73 years, with only 5% of patients under 60. Furthermore, bother may be influenced by patients’ pre-treatment expectations, shaping their perception of symptoms, rather than their actual severity of sexual dysfunction (19). In our investigation, patients were educated before treatment about the temporary nature of testosterone suppression and gradual sexual recovery with relugolix.

Recent research findings point to a potential gap in patient education regarding the side effects of ADT on sexual function. Kinnaird et al. discovered that advanced prostate cancer patients are less likely to receive counseling on sexual dysfunction and identified a lack of consensus over the responsibility of UK-based physicians in managing sexual impairment (21). Nevertheless, there is inconsistency in referring patients to sex therapists and psychosexual counseling (22).

The assessment of sexual bother in prostate cancer patients is of significant importance. Sexual bother has been found to positively correlate with depressive symptoms (23). Men who report increased sexual distress tend to experience greater difficulty with relationship adjustment (19). Contrary to the common assumption that sexual dysfunction and bother have a negative impact on the quality of patients’ relationships, studies have shown that individuals with greater sexual bother still report high relationship satisfaction (20, 21). Some plausible reasons for this include increased open communication between partners, flexibility in exploring other forms of intimacy, and shared efforts in overcoming barriers in prostate cancer treatment together.

When counseling patients on the implications and side effects of ADT, it is imperative to discuss sexual dysfunction and possible management options. Treatment strategies include medical management with PDE-5 inhibitors, inflatable penile prostheses, vacuum erection devices, and psychological counseling and education (17). One pilot ADT education program aimed to teach both patients and their partners strategies for navigating ADT side effects (24). Participants reported increased self-efficacy after program attendance, and study findings proposed that this improved self-sufficiency may reduce patients’ side effect burden (24). However, it is worth noting that there is wide variation in success rates with ED aids (25). It has also been reported that up to half of prostate cancer patients discontinue erectile aids while receiving ADT (26). While further research is needed to elucidate these observations, erectile function is just one component of overall sexual function, and ideal management of sexual side effects is likely multimodal.

Limitations to our study include its small sample size and lack of longitudinal follow-up. The constraints of our sample size made it challenging to conduct sub-analyses and explore relevant variables of interest such as age. As opposed to prior studies that followed patients for an extended time period, our follow-up was also relatively short. Despite these limiting factors, our investigation remains one of the first to specifically examine the effects of relugolix on sexual function.

## 5 Conclusions

In patients with intermediate to high-risk prostate cancer, neoadjuvant relugolix was associated with a significant decline in self-reported sexual function, including erection quality and frequency, orgasm, and overall sexual function. Interestingly, relugolix did not appear to significantly increase sexual bother. Further investigations should compare relugolix to GnRH agonists and evaluate for possible differences in sexual side effect profiles. Another future direction for the study is to analyze changes in sexual functioning and bother over a longer follow-up period to examine how testosterone recovery impacts patients’ self-reported sexual impairment.

## Data availability statement

The raw data supporting the conclusions of this article will be made available by the authors, without undue reservation.

## Ethics statement

The studies involving humans were approved by MedStar Georgetown University Hospital IRB. The studies were conducted in accordance with the local legislation and institutional requirements. The participants provided their written informed consent to participate in this study.

## Author contributions

JH: Conceptualization, Data curation, Formal analysis, Investigation, Writing – original draft, Writing – review & editing. LG: Conceptualization, Data curation, Formal analysis, Investigation, Writing – original draft, Writing – review & editing. MK: Data curation, Formal analysis, Writing – review & editing. SE: Data curation, Formal analysis, Writing – review & editing. SaS: Writing – review & editing. MW: Writing – review & editing. MD: Writing – review & editing. AZ: Writing – review & editing. MA: Writing – review & editing. DK: Writing – review & editing. PL: Writing – review & editing. ND: Writing – review & editing. SiS: Writing – review & editing. RR: Writing – review & editing. SC: Conceptualization, Data curation, Formal analysis, Funding acquisition, Investigation, Methodology, Project administration, Resources, Supervision, Validation, Writing – review & editing.
